# Scientific misconduct and accountability in teams

**DOI:** 10.1371/journal.pone.0215962

**Published:** 2019-05-02

**Authors:** Katrin Hussinger, Maikel Pellens

**Affiliations:** 1 University of Luxembourg (Luxembourg), Faculty of Law, Economics and Finance, Luxembourg, Luxembourg; 2 KU Leuven, Department of Managerial Economics, Strategy and Innovation, Leuven, Belgium; 3 Centre for European Economic Research (ZEW), Department of Economics of Innovation and Industrial Dynamics, Mannheim, Germany; 4 Ghent University, Department of Marketing, Innovation and Organisation, Ghent, Belgium; University of Padova, ITALY

## Abstract

Increasing complexity and multidisciplinarity make collaboration essential for modern science. This, however, raises the question of how to assign accountability for scientific misconduct among larger teams of authors. Biomedical societies and science associations have put forward various sets of guidelines. Some state that all authors are jointly accountable for the integrity of the work. Others stipulate that authors are only accountable for their own contribution. Alternatively, there are guarantor type models that assign accountability to a single author. We contribute to this debate by analyzing the outcomes of 80 scientific misconduct investigations of biomedical scholars conducted by the U.S. Office of Research Integrity (ORI). We show that the position of authors on the byline of 184 publications involved in misconduct cases correlates with responsibility for the misconduct. Based on a series of binary regression models, we show that first authors are 38% more likely to be responsible for scientific misconduct than authors listed in the middle of the byline (p<0.01). Corresponding authors are 14% more likely (p<0.05). These findings suggest that a guarantor-like model where first authors are ex-ante accountable for misconduct is highly likely to not miss catching the author responsible, while not afflicting too many bystanders.

## Introduction

Over the course of the 20^th^ century, science evolved from a norm of single authorship to collaboration. The average number of co-authors per publication grew from almost zero to between 2 and 7 at the end of the century, depending on the discipline [1]. 99.4% of science and engineering subfields exhibited an increase in average team sizes between 1955 and 2000, while team-based publications receive more citations than single-authored publications in 97.7% of subfields [2]. Growing complexity and multidisciplinarity as well as harsh performance evaluation policies render teamwork indispensable [1–4]. This raises normative questions about the distribution of credit and accountability among team members [5,6]. As more collaborators take part in a project, it becomes more difficult to negotiate who should and should not receive credit, and who should be held accountable for individual contributions and the integrity of the final work.

This paper considers a particular aspect of teamwork: who to hold accountable for scientific misconduct. As association with misconduct has severe implications for even loosely associated individuals [7], it is important to design accountability rules that maximize the chances of identifying misconducting scientists, but also to limit the chance of involving scientists who are not guilty of misconduct. Approaches to accountability that are too broad might raise the cost of working with potentially bad or even hard to assess collaborators to such an extent that scientists might choose not to collaborate [8].

There are a number of different approaches to author accountability. Based on the previous literature [8–11], we found four major scientific societies whose authorship guidelines include accountability regimes which are relevant for biomedical scholars. Guidelines by the International Committee of Medical Editors (ICMJE) and the Council of Science Editors (CSE) specify that authors are only directly accountable for the work they contribute. The Committee on Publication Ethics (COPE) and ALL European Academies (ALLEA) hold all authors accountable for the integrity of the complete work, unless otherwise specified. We refer to the former as ‘individual accountability’, and to the latter as ‘joint accountability’.

Additionally, a number of scholars have proposed guarantor type models which suggest allocating accountability to one principal author-guarantor who takes final accountability for the integrity of the entire work. Other authors are listed as contributors together with their contributions [9,12–15]. Guarantors made substantial contributions to the research but also made efforts to verify and uphold the integrity of the study [9]. Due to the gravity of taking on full accountability, guarantors would often be one of the principal authors. Principal authors are those who “take direct responsibility for the manuscript. In addition, they often direct or manage the conduct of the research project, and serve as guarantors of the integrity of the study.” Principal authorship would often correspond to first authorship [15]. In line with this, the American Psychological Association suggests that first authors should act as a guarantor for the study [16]. Whereas guarantor type models have been proposed since the late 90s, their introduction into practice is a recent phenomenon. A growing number of journals now also requires authors to include contributor statements [17].

*Which guidelines are most commonly applied*? To the best of our knowledge, there are no studies systematically comparing the usage of the authorship guidelines listed here. The ICMJE guidelines have been described as the ‘best known and most influential authorship criteria’ [10]. Analysis of a random sample of 234 biomedical journals revealed that, of the journals providing guidelines (59%) 50.7% cited the ICMJE guidelines, 24.6% used other criteria, and 24.6% simply required that all authors approve the manuscript [18]. This corroborates other studies that affirm that “less than half [of top-ranking peer-reviewed journals] described procedures for handling allegations of misconduct.” [19].

Looking at membership to the societies provides an alternative approximation of the application of specific guidelines. Table 1 shows that ICMJE has the widest membership, with ca. 4,500 journals having requested to be listed. CSE has distinctly fewer members, less than 700. With regard to the joint accountability guidelines, COPE sports a strong 12,000 member journals, about 3,000 of which are in the field of medicine. Finally, ALLEA does not report member journals, but claims to be supported by approximately 60 member academies and learned societies. The uptake of guarantor models is still modest, although they have been adopted by, among others, the American Psychological Association [16] and the BMJ [20]. Somewhat more broadly, 11 out of the 15 top journals in the multidisciplinary sciences now require contribution disclosures [17].

**Table 1:** Indicative prominence of authorship guidelines.

Guidelines	Indicative use
ICMJE	Approx. 4,500 journals asked to be listed
CSE	Approx. 690 member journals
COPE	Approx. 12,000 member journals, of which 3,000 in medicine
ALLEA	60 member academies and learned societies

Indicative use of authorship guidelines, as reported by organizations. Reflects situation in July 2018.

Sources: ICMJE:http://www.icmje.org/journals-following-the-icmje-recommendations/; CSE:www.councilscienceeditors.org/about/members-journals; COPE:publicationethics.org/members ALLEA:www.allea.org/about-allea.

*How do the approaches compare*? Joint accountability has been criticized as unreasonable and unrealistic, especially in the context of growing author teams [8,12,21]. Team work has proliferated precisely because of increasingly complex research challenges that require specialization to solve them. Therefore, holding all authors accountable for all contributions seems unfair, is costly and creates disincentives to collaboration. Kempers cites the example that “*the clinician who stages the cancer and collects the specimens cannot vouch for the analysis by the molecular biologist nor for the analysis of the data by the statistician*” [21]. Rennie argues that “*because the only reason you seek coworkers is that they have skills you yourself lack*, *it is delusional and dangerous to expect you to answer for that which by definition you cannot know enough about because you have from the start handed off responsibility for it to another”*[12]. Regardless of skill, it seems morally unjust to hold scientists responsible for tasks they did not perform: how can it be just to hold the scientist who wrote the literature review responsible for data manipulation performed in the lab, a task in which he was not involved? Helgesson and Eriksson conclude that joint accountability is not acceptable from a moral point of view, as it entails treating innocent researchers as if they were guilty of misconduct if the case should arise. Such practices might lead scientists to be become demotivated when they feel mistreated, and might increase the cost of picking a bad collaborator to such an extent that researchers become hesitant towards embarking on new collaborations [8].

At first glance, joint accountability might seem attractive from an economic perspective. It is difficult and costly to identify the researcher ultimately responsible for scientific misconduct. For instance, an in-depth analysis of a misconduct investigation yielded an estimate of more than $500,000 spent on legal and consulting fees, faculty and witness salary, and other expenses [22]. When detection is so costly, group punishment might appear to be more efficient. However, this comes at the high cost of harming innocents [23,24]. Social costs are particularly high in this setting, as scientific misconduct stigmatizes even loosely associated individuals, leading valuable research results to be ignored as the academic community distrusts scientists in the vicinity of misconduct [7].

We contribute to the debate on accountability by analyzing who is responsible for scientific misconduct within author teams. Concretely, we assess whether the results of U.S. Office of Research Integrity (ORI) investigations correspond best with existing accountability guidelines or a guarantor model. By doing so we rely on the critical distinction between responsibility and accountability [8]. Responsibility implies moral blameworthiness and often direct culpability. That means, the responsible person is the one who actually committed the scientific misconduct. Accountability entails ‘being the one to blame when things go wrong’ [8].

Our analysis is based on a set of 184 publications in biomedical science from past investigations of 80 misconduct cases conducted by the ORI. We estimate Probit models of being responsible for misconduct within the author team as a function of the author’s position on the byline. This reflects relative contribution and credit: first, senior (last), and corresponding authors contribute more, and receive more credit, than contributing (middle) authors [21,25–27]. We find that first authors are 37% more likely to be found responsible for scientific misconduct than middle authors. Corresponding authors are 12% more likely.

This observation informs the design of accountability guidelines; guidelines requiring authors to share accountability do not reflect the fact that first authors and corresponding authors are most likely to actually be responsible for misconduct. This means that the social cost of wrongfully holding contributing authors accountable for misconduct is likely higher than its benefits. Guarantor models should instead prove to be more efficient, to the extent that they allocate accountability to authors with a central role.

## Materials and methods

### Data

The main data source for this project is the population of Findings of Scientific Misconduct published by the Office of Research Integrity (ORI). The ORI directs activities concerning research integrity within the Public Health Services (PHS), including handling and overseeing investigations of scientific misconduct in research funded by the National Institutes of Health (NIH) and the PHS. The ORI either directly pursues inquiries and investigations into allegations of scientific misconduct, or oversees institutions pursuing independent actions. When evidence of scientific misconduct surfaces, details about the misconduct case, including who was involved, what transpired, any affected publications, and sanctions taken are published in Findings of Research Misconduct as well as annual reports.

The transparent reporting of misconduct by the ORI provides a major advantage, compared to other data sources such as retractions: there is no ambiguity regarding the person responsible for misconduct within coauthor teams. This data thus allows us to compare, within retracted or corrected publications authors who are responsible for misconduct with those who are not. Prior studies have used this data to analyze other aspects of scientific misconduct such as different types of accusations and outcomes, sources of funding, trends over time, effects on innocent bystanders etc. [7,28–34].

For the purpose of our analysis, we focus on the complete list of 232 misconduct cases published between 1993 and 2014. Of these, 81 involved at least one retracted or corrected publication, for a total of 227 affected publications. For 196 of these publications, bibliographic details could be retrieved on Elsevier’s Scopus bibliometric database. Eleven publications were dropped because their publication date was in years with too few publications in the sample to adequately estimate year effects. These are publications earlier than 1985, from 1987, and from 2012. We additionally drop one case where the misconducting scientist was not part of the author team. The final sample covers 80 misconduct cases and 184 publications.

**Fig 1:**
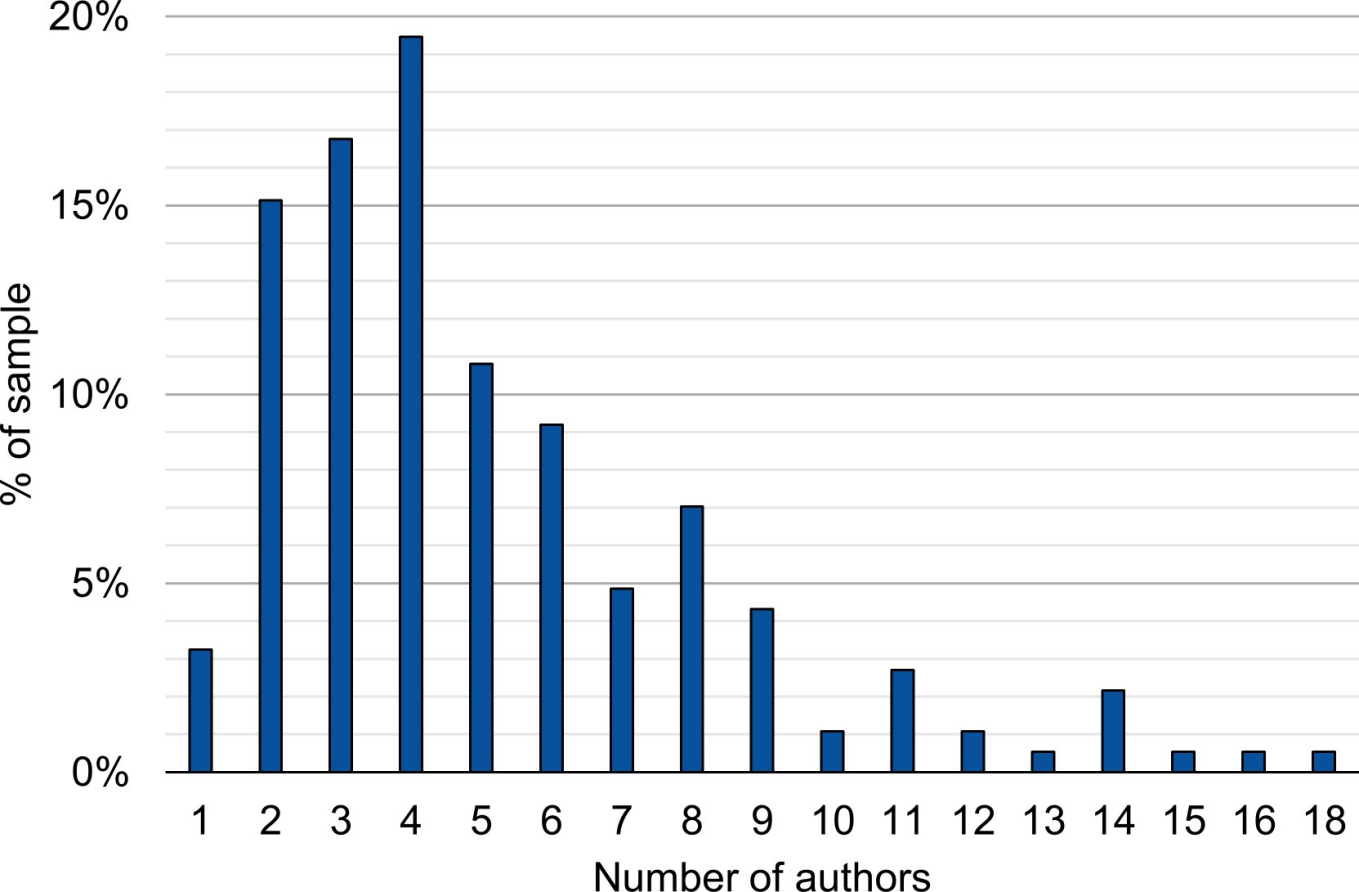
Number of authors per article in sample.

Fig 1 shows the distribution of the number of coauthors per article. The median publication in the sample has 4 authors; the mean is 5.17. Only 6 articles are single-authored. The tail of the author distribution is long, with up to 18 authors documented in the sample.

We then collected the publication data of all authors listed on the 184 publications in the final sample. The final dataset is on the publication-author level and contains 951 publication-author 1:n relations. For every publication, all authors are listed, along with information about whether they were deemed responsible by the ORI, their role in the publication, and their bibliometric profile at the time of the article’s publication.

*External validity of the sample*. The number of authors per publication in our sample is highly similar to those reported by Fanelli and Larivière for a broader sample of biomedical scientists [1]. However, the key question is not whether our data is representative for biomedical science as a whole, but whether it is representative for science in which misconduct takes place. Whereas it is not possible to know whether the data is representative for the full, largely undiscovered, population of publications containing misconduct, our data is based on the entire population of misconduct cases reported by the ORI.

### Variable definitions

In order to analyze which accountability regime fits best to the empirical evidence for responsibility, we estimate the likelihood of being the author responsible for scientific misconduct within an author team. The dependent variable is a binary variable that captures whether the respective author was deemed guilty of misconduct by the ORI, in other words, whether or not the author was factually responsible for committing misconduct (*RESP*).

The main variables of interest capture the author’s position on the byline of the affected publications. A rich literature studies the meaning of author order in biomedical disciplines [12,15,21,25,26,35]. The most important author, in terms of contribution as well as credit, is the first author. The first author did the majority of the experimental work and takes credit as originator of the idea. Being first author is such an important precondition for being accepted as scientist that many PhD programs require students to publish at least one first-author article before graduation [25]. The next most important position is that of the senior author, who is usually listed last. This position is one of more general oversight and direction. Senior authors also have an important role guaranteeing the authenticity of the work reported. Another important role is for the corresponding author, the author responsible for communicating about the publication with editors and readers. This position typically coincides with that of the first or senior author [25], but can also be used as a tool for sharing credit when authors other than the first or senior should be credited more than usual. Least credit goes to middle, or contributing authors, who are listed between the first and senior author. Their order reflects their relative contribution, which is significantly less than the contributions of either the first or senior author. In some cases, the second author is seen as the second-most important author [21,35]. Author positioning has also been found to correlate positively with the amount of contribution [17] and are determined by effort, not prestige or position [35].

In line with this literature, we distinguish between first authors (*FIRST*) and senior (last) authors (*SENIOR*). We further divide contributing authors in second authors (*SECOND*) and others (*MIDDLE*), to take in to account that the former might receive more credit. If the relevant publication was single-authored, the author was coded as first author. If there were two co-authors, the second one was labeled as the senior author, and not as second author. We also take into account which author was marked as corresponding author on the publication (*CORR*). Additionally, we record the number of authors on the focal paper (*NAUTHORS*) and the article’s publication year.

### Methods

We are interested in the relationship between author position and responsibility for scientific misconduct. The analysis starts with a descriptive comparison of the proportion of responsible and not responsible authors across roles. However, such comparison might over- or underestimate the true relation between author position and responsibility because there might be other variables which correlate with both the roles authors take on in scientific teams and the likelihood that they commit misconduct. In the analysis, we will therefore employ multivariate regression analysis to estimate the probability that a given author was found guilty of scientific misconduct depending on the author’s position on the byline. This allows us to control for correlations between author roles (i.e. first authors and senior authors cannot occur in the same observation, so they correlate negatively), team size, and joint year effects. Joint year effects reflect potential changing incentives to engage in misconduct over time. We include team size in the regression model to account for the fact that the baseline probability of being responsible decreases as the number of authors increases (as there is one responsible author per publication). As our sample consists of sets of coauthors linked to corrected and retracted publications, they likely violate the assumption that observations are i.i.d. Therefore, we calculate standard errors clustered by misconduct case.

We employ a Probit regression model, specified as P(*RESP* = 1|*X* = *x*) = Φ(*βX*), where Φ is the cumulative distribution function of the standard normal distribution. *β* is a vector of parameters to be estimated, and *X* represents a set of variables. Logit models and linear probability models estimated through OLS regression yield highly similar results. *X* includes our explanatory variables, that is, the author position variables *FIRST*, *SECOND*, *SENIOR*, and *CORR*, as well as *NAUTHORS*. *MIDDLE* forms the base category of authorship position and is therefore excluded. We also include a full set of publication year effects in order to capture joint shocks in the likelihood of *RESP*, for instance through joint trends in typical author team size. We estimate four main models, each of which adds variables to the analysis. The first model takes the form

$$P(RESP=1)=\Phi (\alpha +\beta_{1}FIRST+\beta_{2}SECOND+\beta_{3}SENIOR+\epsilon )$$ (1)

Here, *β*_1−3_ are key coefficients to be estimated, and represent the difference in probability of being responsible for misconduct as compared to a middle author (the reference category). *α* is a shared constant and *ϵ* is the error term. In a second model, we add *CORR* to the model to account for the fact that authorship credit and corresponding authorship are intertwined with one another as well as with the responsibility for the work:

$$P(RESP=1)=\Phi (\alpha +\beta_{1}FIRST+\beta_{2}SECOND+\beta_{3}SENIOR+\beta_{4}CORR+\epsilon )$$ (2)

In a third model, we add controls for the number of authors of the focal publication (*NAUTHORS*) and a set of year indicators to capture joint trends (*δ*_*t*_). The model then becomes:

$$P(RESP=1)=\Phi (\alpha +\beta_{1}FIRST+\beta_{2}SECOND+\beta_{3}SENIOR+\beta_{4}CORR+\beta_{5}NAUTHORS+\delta_{t}+\epsilon )$$ (3)

It might also be the case that in some particular cases the ORI does not identify the scientist who is actually responsible. To further test whether our results are robust to violating the key assumption that the ORI catches the responsible author, we estimate an additional model (model 4) where we exclude cases where the ORI reported that the responsible scientist denies the ORI’s conclusion and or appealed the ORI’s decision. This was the case for 25% of the cases in the sample.

We performed additional analyses including author experience (time since first publication) as well as reputation (citations stock), both scientist characteristics that can influence the incentive to engage in misconduct, as additional control variables. While there is evidence that scientists’ eminence and early career scientists are more likely to engage in scientific misconduct as they have more to gain and less to lose [36,37], the variables turn out to have limited explanatory power in our specific analysis and the main results do not change. They are available in the supplementary program code (see S1 Data and S1 Code) or upon request from the authors.

## Results

### Descriptive Statistics

**Fig 2:**
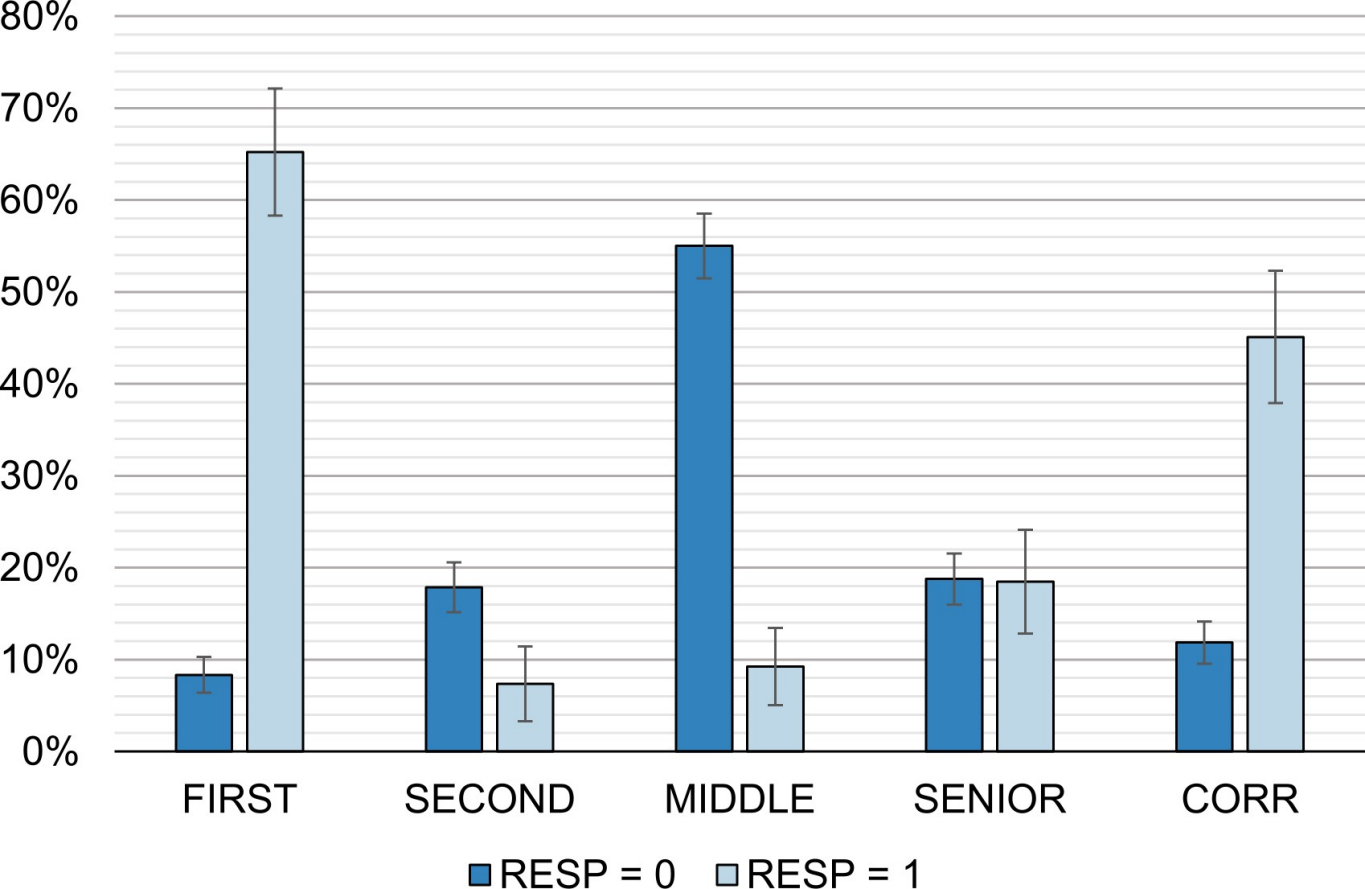
Author position by responsibility for misconduct. The graph shows the proportion of responsible and not responsible authors in each author position. The first four bars (*FIRST*, *SECOND*, *MIDDLE*, *SENIOR*) add to 100% as each author is assigned one position. Note that the dark bars only add up to 99% due to rounding. One of the positions is assigned corresponding authorship. Among responsible authors, there is a significantly larger proportion of first authors and corresponding authors, and a smaller proportion of second and middle authors. Senior authors are represented similarly in each group. Error bars indicate 95% confidence intervals.

Fig 2 shows the distribution of observations across author positions. The authors responsible for misconduct are primarily first authors (65% of responsible vs. 8% of not responsible; test of equal proportions: *χ*^2^(1) = 303.97; p<0.0001). Likewise, a larger share of responsible authors is corresponding author (45% vs. 12%; *χ*^2^(1) = 107.50; p<0.0001). Senior authors account for a similar share of responsible and not responsible authors (18% of responsible vs. 19% of not responsible; *χ*^2^(1) = 0.003; p = 1.000). Second and middle authors are, however, less present among responsible authors and more among innocent authors (*SECOND*: 7% of responsible vs. 18% of not responsible; *χ*^2^(1) = 12.22; p = 0.0005; *MIDDLE*: 9% of responsible vs. 55% of not responsible; *χ*^2^(1) = 123.31; p<0.0001). In the remainder of the analysis, we perform regression analysis to verify the robustness of these relationships to controlling for author’s characteristics.

### Multivariate analysis

**Fig 3:**
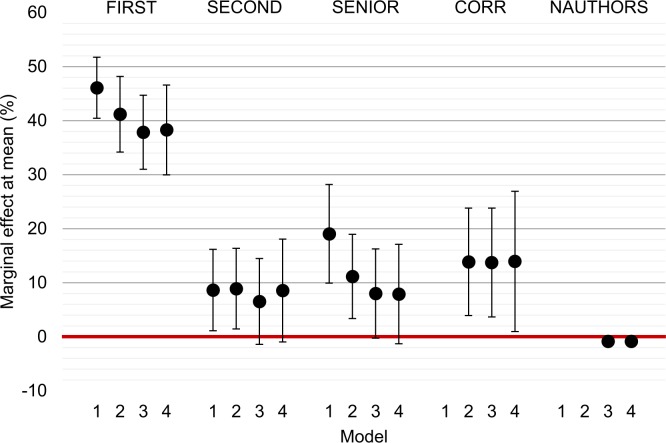
Coefficient plot of Probit regression of author position on responsibility. The dots indicate the predicted marginal effect of the model variables on the probability of being responsible for scientific misconduct at the sample mean. The error bars indicate 95% confidence intervals of the marginal effect. The underlying model is a Probit regression model. Standard errors are clustered by misconduct case. The estimates are grouped by model. Model one includes FIRST, SECOND, SENIOR, and a constant. The coefficients represent the added probability of being found responsible compared to middle authors, the reference group. Model two includes a variable for corresponding authorship, CORR. Model three controls for the number of authors on the publication (NAUTHORS) and includes a set of year indicators. Model four follows the same specification as model three but limits the sample to those where the responsible author did not deny or appeal ORI’s findings. Across all models, first authors remain significantly more likely to be responsible than middle authors. Senior authors are also more likely to be responsible in model one and two, but this effect turns insignificant once CORR is added to the analysis. Note that the 95% confidence intervals for NAUTHORS are small: [-1.3%—-0.4%] for both models where they are included.

Fig 3 shows the results of the regressions by plotting the marginal effects at the mean implied by the estimated coefficients. See S1 Table for the detailed regression results.

Model 1 only includes the author position indicators *FIRST*, *SECOND*, and *SENIOR* as explanatory variables. First authors as well as senior authors are significantly more likely to be found responsible for misconduct than middle authors with marginal effects of respectively 46.1 and 19.0 percentage points at the mean (FIRST: 95% CI: 40.4–51.7, p < 0.001; SENIOR: 95% CI = 9.9–28.2, p < 0.001). Second authors are also more likely to be responsible, but the effect is smaller at 8.6 percentage points (95% CI: 1.1–16.1, p = 0.025). This is probably because second authors are in many cases considered to be no different than other middle authors.

Model 2 includes *CORR* as additional indicator of author credit. The coefficient is positive and significant with a marginal effect at mean of 13.8 percentage points (95% CI: 3.9–23.8, p = 0.006). The coefficients of FIRST and SENIOR drop to respectively 41.2 percentage points (95% CI: 34.2–48.2, p < 0.001) and 11.2 percentage points (95% CI: 3.4–18.9, p = 0.004).

Model 3, our preferred model, adds the number of authors on the publication (*NAUTHORS*) to the model to account for the fact that the ex-ante probability of being responsible for misconduct within an author team decreases in the number of coauthors, as well as a set of calendar year indicators. The effect size of first authors drops somewhat, to 37.8 percentage points (95% CI: 31.0–44.7, p<0.001). Corresponding authors maintain an estimated positive coefficient at 13.7 percentage points higher probability (95% CI: 3.6–23.8, p = 0.008). Senior authors are in this model not statistically more likely to be found responsible than middle authors (mfx at mean: 8.0 percentage points, 95% CI: -0.3–16.2, p = 0.058), as are second authors (mfx at mean: 6.5 percentage points, 95% CI: -1.4–14.4, p = 0.108). The coefficient of NAUTHORS is negative, as expected. Conditional on the other variables, every additional author decreases the baseline probability of being responsible for misconduct with 0.9 percentage points (95% CI: -1.3 - -0.4, p<0.001).

Model 4 reports the robustness check excluding cases where the responsible author denied responsibility or appealed the case. As this model leads to highly similar conclusions as the full sample, we exclude that our previous conclusions were driven by misallocated responsibility for misconduct.

## Discussion and conclusion

The analysis shows that scientific misconduct is not equally likely to arise from all members of an author team. First authors, corresponding authors, and to some extent senior authors are much more likely than middle authors to be found responsible for scientific misconduct in Findings of Research Misconduct by the U.S. Office of Research Integrity. Authorship policies that impose accountability for the integrity of all research findings on the shoulders of all coauthors miss this important fact. Our findings make intuitive sense. Those who have most to gain from the work in terms of scientific credit arguably have the highest incentives to commit scientific misconduct. While middle authors will do equal harm to their careers if found guilty of scientific misconduct, they reap much less of the reward since they are not lead author of the projects.

The fact that middle authors are strongly underrepresented amongst misconducting scientists aggravates the high social costs of group punishment [23,24]. The cost of false punishment is particularly nefarious in the context of scientific misconduct, and deeply affects scientists’ career prospects [7]. We agree with the view of Helgesson and Eriksson [8]: punishing innocent coauthors based on transgressions they did not do cannot be morally just unless a strong case can be made that coauthors implicated themselves through omission. This, however, should be proven, not assumed. Internal monitoring is also unlikely to be fully effective given the ever increasing degree of specialization within author teams [12,21]. Moreover, incorporating the prior knowledge that misconduct is less likely to stem from middle authors, ex ante, would even lower the potential cost of misconduct investigations through increased efficiency.

What is the best accountability policy? The goal of accountability policy should be to shift accountability to those who are most likely to commit misconduct while not unnecessarily implicating innocent coauthors. This is of course only true ex ante; if one author, through investigation, is found to be responsible for the misconduct, this author should be held accountable for it. Others should only be implicated to the extent that they violated ethical norms themselves through omission. The finding that first and senior authors are much more likely to commit misconduct is indicative that the shared accountability imposed by the COPE and ALLEA guidelines lays too much accountability at the feet of contributing authors (one should acknowledge, however, that both leave room for authors to not take responsibility for certain parts of the work). The policies by ICMJE and CSE, compared to COPE and ALLEA, seem to impose distinctly lower social costs in terms of potential stigmatization in cases of misconduct through their approach of individual accountability. Better still, however, might be a guarantor-type model [9,12–15]. Perhaps the best option, in terms of matching accountability principles to empirical reality, would be a guarantor type model that holds principal authors ex ante accountable for misconduct, given that they have the best knowledge of the essence of the project and the highest incentives to commit or to prevent misconduct.

## Supporting information

S1 Table Probit regression of responsibility for misconduct. This table contains the full regression results used to generate Fig 3.
(PDF)

S1 Data Reproduction data. This file contains reproduction data in CSV format. The data is on the publication-author level and contains the following information. *CASE* is an index of ORI misconduct cases. *INDEX* is an index of affected publications. *AUTHORNUMBER* is the author number of the relevant author. *NAUTHORS* contains the total number of authors listed on the publication. *CYEAR* is the publication year of the article. *RESP* takes value 1 if the focal author was found responsible for misconduct by the ORI, and 0 otherwise. *DENIALCASE* takes value 1 if the ORI findings reported that the responsible scientist denies the ORI’s conclusion and or appealed the ORI’s decision. *DENIALSAMPLE_EXCLUDE* takes value 1 for publication years that have too few observations to properly estimate year fixed effects once observations with *DENIALCASE* equal to 1 are excluded, and 0 otherwise. These publication years are eliminated from the sample in model 4 of Fig 3 and S1 Table. *AUTH_FIRST*, *AUTH_SECOND*, *AUTH_SENIOR*, *AUTH_MIDDLE* take value 1 if the author is first, second, senior, or middle author, respectively, and 0 otherwise. *AUTH_CORR* takes value 1 if the author is indicated to be corresponding author, and 0 otherwise. *CAREERAGE* contains the number of years since the author’s first publication at the article’s time of publication. *CAREERAGESQ* contains the same information squared. *LNCITEDBYSTOCK* contains the natural logarithm of the perpetual inventory stock of citation-weighted publications that the author published up to the year in which the focal article was published (depreciation rate: 15%).
(CSV)

S1 Code Reproduction code. This file contains R code that reproduces the findings presented in Figs 1–3 and S1 Table.
(R) Click here for additional data file.
