# Supplementary material for: Scientific misconduct and accountability in teams
Source: PLoS One. 2019 May 2;14(5):e0215962. doi: 10.1371/journal.pone.0215962 (PMC6497379; doi:10.1371/journal.pone.0215962)
Supplement: S1 Table — This table contains the full regression results used to generate Fig 3. (PDF) [file pone.0215962.s001.pdf]

S1 Table. Probit regression of responsibility for misconduct.

	Dependent variable: RESP			
	Full Sample			*Excluding denial & appeal*
	(1)	(2)	(3)	(4)
FIRST	2.157***	1.953***	1.816***	1.791***
	(0.194)	(0.202)	(0.210)	(0.247)
SECOND	0.404**	0.422**	0.313	0.400
	(0.184)	(0.185)	(0.198)	(0.234)
SENIOR	0.892***	0.529***	0.383	0.369
	(0.230)	(0.191)	(0.205)	(0.223)
CORR		0.657***	0.659**	0.652**
		(0.254)	(0.257)	(0.320)
NAUTHORS			-0.042***	-0.041***
			(0.009)	(0.011)
Constant	-1.766***	-1.794***	-1.468***	-1.298***
	(0.103)	(0.109)	(0.175)	(0.233)
Observations	951	951	951	669
Publications	184	184	184	132
Cases	80	80	80	60
Publication year FE	NO	NO	YES	YES
Mfx. at mean				
FIRST	46.1%	41.2%	37.8%	38.3%
SECOND	8.6%	8.9%	6.5%	8.6%
SENIOR	19.0%	11.2%	8.0%	7.9%
CORR		13.8%	13.7%	13.9%
NAUTHORS			-0.9%	-0.9%

Probit regression model. Standard errors are clustered by misconduct case. The dependent variable is whether the author is responsible for misconduct. The key explanatory variables are author position (FIRST, SECOND, SENIOR; MIDDLE is base category) and corresponding author status (CORR). Model one includes FIRST, SECOND, SENIOR, a set of year indicators, and a constant. Model two adds CORR. Model three adds controls for NAUTHORS and joint year effects. Model four follows the same specification as model three but limits the sample to those where the responsible author did not deny or appeal ORI's findings. Standard errors clustered by misconduct case.

Stars indicate p-value of coefficient significance test:

*** Significant at the 1 percent level.

** Significant at the 5 percent level.
